# Supplementary material for: Cyclone vulnerability assessment of the central coast of Bangladesh: A comprehensive study utilizing FAHP and geospatial techniques
Source: PLoS One. 2025 Jul 9;20(7):e0326965. doi: 10.1371/journal.pone.0326965 (PMC12240350; doi:10.1371/journal.pone.0326965)
Supplement: Attachment — Submitted filename: Response to Reviewers.docx [file pone.0326965.s002.docx]

**Manuscript Title:** Cyclone vulnerability assessment of the central coast of Bangladesh: a comprehensive study utilizing FAHP and geospatial techniques

**Authors:** Md. Abdullah Salman^1*^, Mahmudul Hasan Rakib^2^, Shacin Chandra Saha^2^, Md Emdadul Haque^2^, Md Sabbir Hossen^2^

Journal Name: PLOS ONE

Manuscript ID: **PONE-D-24-47688**

Dear Editor & [Reviewer],

Thank you for your thorough review and constructive feedback on our manuscript. We appreciate the time and effort you dedicated to improving the quality of our work.

We have carefully considered all comments and suggestions. Below, we provide a point-by-point response to each comment. Revisions made to the manuscript are highlighted in yellow for clarity.

**Journal requirements:**

**Comment 01:**

Please ensure that your manuscript meets PLOS ONE's style requirements, including those for file naming. The PLOS ONE style templates can be found at <https://journals.plos.org/plosone/s/file?id=wjVg/PLOSOne_formatting_sample_main_body.pdf> and <https://journals.plos.org/plosone/s/file?id=ba62/PLOSOne_formatting_sample_title_authors_affiliations.pdf>

**Response:**

Thank you for bringing this to our attention. We have reviewed the PLOS ONE style requirements and made the necessary adjustments to ensure compliance. Specifically, we have:

1. Revised the manuscript formatting to align with the journal's guidelines.
2. Renamed all files according to the specified file naming conventions (e.g., "Manuscript," "Fig1.tif," "Table1.docx").

**Comment 02:**

Please note that PLOS ONE has specific guidelines on code sharing for submissions in which author-generated code underpins the findings in the manuscript. In these cases, we expect all author-generated code to be made available without restrictions upon publication of the work. Please review our guidelines at <https://journals.plos.org/plosone/s/materials-and-software-sharing#loc-sharing-code> and ensure that your code is shared in a way that follows best practice and facilitates reproducibility and reuse.

**Response:**

Thank you for highlighting the importance of code sharing. Due to legal/ethical reasons, such as confidentiality agreements, intellectual property restrictions, or patient data privacy concerns, we are unable to make the code publicly available.

To ensure transparency and accessibility within these constraints:

1. **Data Availability Statement Update:** All data are in the manuscript itself

The add changes can be found on page 21, lines 810–811.

**Comment 03:**

In your Methods section, please provide additional information regarding the permits you obtained for the work. Please ensure you have included the full name of the authority that approved the field site access and, if no permits were required, a brief statement explaining why.

**Response:**

No specific permits were required for the work described in this study, as it was conducted in publicly accessible areas. The field site access complied with all applicable local, national, and international regulations. The changes can be found on page 5, lines 196–198.

**Comment 04:**

Please provide a complete Data Availability Statement in the submission form, ensuring you include all necessary access information or a reason for why you are unable to make your data freely accessible. If your research concerns only data provided within your submission, please write "All data are in the manuscript and/or supporting information files" as your Data Availability Statement.

**Response:**

To ensure transparency and accessibility within these constraints:

- **Data Availability Statement Update:** All data are in the manuscript itself

The add changes can be found on page 21, lines 810–811.

**Comment 05:**

We note that Figures 1, 3A-E, 4A-G, 5A-F, 6, 7, 8 in your submission contain [map/satellite] images which may be copyrighted. All PLOS content is published under the Creative Commons Attribution License (CC BY 4.0), which means that the manuscript, images, and Supporting Information files will be freely available online, and any third party is permitted to access, download, copy, distribute, and use these materials in any way, even commercially, with proper attribution.

**Response:**

Thank you for raising this concern. The map/satellite images used in Figures 1, 3A-E, 4A-G, 5A-F, and 6A-C, were obtained from publicly available sources [USGS EROS (Earth Resources Observatory and Science (EROS) Center) (public domain): [http://eros.usgs.gov/#](http://eros.usgs.gov/)] that are explicitly designated as being in the public domain. These images are not subject to copyright restrictions, and appropriate attribution. If further clarification or documentation is needed, we would be happy to provide it. These changes can be found on page 05, “Datasets” section and “Table 1”. **Figure captions have mentioned the courtesy of USGS.**

**Additional Editor Comments:**

**Comment 01:**

The abstract could benefit from a more streamlined presentation. Some sentences are slightly repetitive, particularly regarding vulnerability factors, and could be combined for brevity. The abstract could more explicitly highlight the practical implications of the study's findings, particularly in terms of how the results could influence policy or lead to tangible improvements in cyclone preparedness.

**Response:**

The updated version (page 02, lines 38-58) reduces repetition, emphasizes practical implications, and maintains the original intent.

**Comment 02:**

"Spatial Distribution of Vulnerability" Section in results and discussion, The section is generally clear but could be structured better for readability. The discussion on physical, social, and mitigation vulnerability seems fragmented. It might help to more clearly separate the findings on each criterion (physical, social, and mitigation), rather than switching between them in the same paragraph.

**Response:**

Thank you for this valuable suggestion. We have revised the "Spatial Distribution of Vulnerability" section to improve its structure and readability. Specifically, we have reorganized the discussion to clearly separate findings related to physical, social, and mitigation vulnerability. Each criterion is now addressed in its own subsection to ensure a more logical flow and to avoid any fragmentation of the discussion. These changes can be found in the revised manuscript on pages [13-17], and we hope this restructuring enhances the clarity and coherence of the section.

**Comment 03:**

When referring to maps and tables, ensure a consistent description (e.g., "Fig 6 illustrates..." followed by "As shown in Table 5...") to improve flow.

**Response:**

Thank you for pointing out the need for consistent descriptions when referring to maps and tables. We have revised the manuscript to ensure a uniform style, using consistent phrasing such as "Fig. X illustrates..." and "As shown in Table Y..." throughout the text. These updates improve the flow and readability of the manuscript. These changes can be found throughout the revised version.

**Comment 04:**

The mention of figures (Fig 6, Fig 7, Fig 8) and tables is helpful but would benefit from a clearer connection between the text and the visuals. It might be more effective to explicitly state what each figure or table illustrates before or after mentioning it.

**Response:**

Thank you for the suggestion. In response to your feedback, we have consolidated Figures 6, 7, and 8 into a single, comprehensive figure (Fig 6A-C) and make a separate section “**Physical, Social Vulnerability and Mitigation Capacity*”*** for clarity and improved presentation. We have also revised the manuscript to explicitly state what this new figure illustrates, ensuring a clearer connection between the text and the visual. The revised figure and the updated text can be found on page 16 [lines 602-625]. We believe this change enhances the overall flow and understanding of the section.

**Comment 05:**

Ensure that the references to the figures and tables are numbered and consistently formatted.

**Response:**

Thank you for highlighting the need for consistent formatting. We have carefully reviewed the manuscript and ensured that all references to figures and tables are correctly numbered and formatted according to the journal's guidelines. We believe this improves the clarity and consistency of the manuscript. These changes can be found throughout the revised version.

**Comment 06:**

The geographical locations mentioned (e.g., Bhola, Borgona, Patuakhali) are important but need further clarification regarding why these areas are specifically vulnerable (e.g., do they have a higher proportion of vulnerable populations, lack of infrastructure, or other unique factors?).

Response:

Thank you for your insightful suggestion. We have expanded the discussion on the specific vulnerability of the geographical locations mentioned (e.g., Bhola, Borgona, Patuakhali) by providing more detailed explanations of the factors contributing to their vulnerability. These areas are particularly vulnerable due to a combination of factors, including a higher proportion of vulnerable populations (e.g., population density, gender, disabled, high dependency on agriculture and vulnerable land covers), limited infrastructure (e.g., inadequate road networks and healthcare systems), and exposure to environmental hazards such as flooding, and cyclones. We have clarified these points in the revised manuscript to provide a more comprehensive understanding of why these regions are specifically at risk. The updated discussion can be found on pages [17-19].

**Comment 07:**

The percentages of the total area mentioned in relation to vulnerability categories are useful, but further explanation of how these figures were derived would enhance credibility. For instance, were these areas assessed based on a specific methodology (e.g., satellite data, field surveys)?

**Response:**

Thank you for your constructive feedback. We have added further details in the manuscript regarding the methodology used to derive the percentages for each vulnerability category. Specifically, these areas were assessed using e.g., satellite imagery, remote sensing data, field surveys, GIS analysis, with FAHP, and field observations. This additional explanation enhances the transparency of our analysis and provides a clearer understanding of how the vulnerability categories and associated percentages were determined. For credibility, we have added a new section “**Validation of cyclone vulnerability”** for showing accuracy levels**.** The updated explanation can be found on page 17 (lines 628-633) of the revised manuscript.

**Comment 08:**

Results and discussion should be separately explained in more detail.

**Response:**

Thank you for your helpful suggestion. We have revised the manuscript to provide a more detailed and clear distinction between the **Results** (start page **13**) and **Discussion** (start page **17**) sections. The Results section now focuses solely on presenting the findings, while the Discussion section has been expanded to include a more in-depth interpretation and analysis of the results. We believe this separation enhances the clarity and structure of the manuscript.

**Comment 09:**

The terms used to describe vulnerability (e.g., “very high,” “high,” “moderate,” etc.) are appropriate, but consistency in terminology is key. Ensure that terms like "susceptibility," "vulnerability," and "sensitivity" are used clearly and consistently across the entire report to avoid confusion.

**Response:**

Thank you for this valuable feedback. We have carefully reviewed the manuscript and ensured that the terms "susceptibility," "vulnerability," and "sensitivity" are used consistently and clearly throughout the report. Any instances of inconsistent terminology have been corrected to avoid confusion and ensure clarity. We believe this revision enhances the precision of the language used in the manuscript. These changes can be found throughout the document.

**Comment 10:**

The use of "moderate" and "moderately vulnerable" seems a bit repetitive, as these terms are used in both physical and social vulnerability contexts. Clarifying how they differ across contexts would improve precision.

**Response:**

Thank you for pointing this out. We have clarified the use of "moderate" and "moderately vulnerable" in the manuscript to use ‘moderate’ term apply in the contexts of physical and social vulnerability. In the context of physical vulnerability, "moderate" refers to areas with a moderate level of exposure to environmental hazards, such as flooding or erosion, while in the social vulnerability context, "moderate" refers to communities that have a moderate ability to adapt to or recover from these hazards due to factors like economic resources or access to social support. We have revised the text to provide clearer explanations of these differences and ensure greater precision. The revisions can be found on pages [13-15] of the updated manuscript.

**Comment 11:**

The data on the physical vulnerability categories (e.g., “high” and “very high” vulnerability accounting for 45% of the area) is helpful, but the underlying factors (e.g., land cover, elevation, proximity to the coast) should be explained in more detail to give readers a clearer understanding of why these factors contribute to vulnerability.

**Response:**

Thank you for your helpful suggestion. We have expanded the explanation of the underlying factors contributing to physical vulnerability, such as land cover, elevation, and proximity to the coast. Specifically, we have clarified how each of these factors influence vulnerability, including their role in determining exposure to hazards (e.g., flooding, cyclone) and the capacity of the environment to withstand or recover from such impacts. This additional context provides readers with a clearer understanding of how these physical factors contribute to the overall assessment of vulnerability. The updated explanation can be found in the revised manuscript on pages [13-14] and lines [483-498].

**Comment 12:**

Similarly, for social vulnerability, while the description of factors such as population density, gender, disability, and agricultural reliance is good, a more detailed discussion on how these factors contribute to vulnerability would strengthen the analysis.

**Response:**

Thank you for your thoughtful feedback. We have expanded the results and discussion of social vulnerability factors such as population density, gender, disability, and agricultural reliance to provide a more detailed explanation of how each of these contributes to overall vulnerability. For example, we now explain how high population density increases exposure to hazards, how gender and disability may affect adaptive capacity and access to resources, and how reliance on agriculture can make communities more vulnerable to environmental shocks. This added context strengthens the analysis and clarifies the relationships between these social factors and vulnerability. The revised discussion can be found on pages [14-17] of the manuscript.

**Comment 13:**

**Mitigation Capacity Discussion**: The section on mitigation capacity could benefit from a more nuanced analysis. While it mentions "poor road networks," "poor cyclone warning systems," and "lack of infrastructure," it would be useful to describe the extent of these deficiencies. For example, how do these issues compare across regions? Are some areas better equipped despite the overall low mitigation capacity in the southern and southeastern regions?

**Response:**

Thank you for this insightful suggestion. We have revised the mitigation capacity section to provide a more nuanced analysis of the deficiencies in road networks, cyclone warning systems, and infrastructure. Specifically, we now include a comparison of how these deficiencies vary across different regions. These regional differences are discussed in more detail to provide a clearer understanding of the variation in mitigation capacity across the study area. The updated analysis can be found in the revised manuscript on pages [15-19].

**Comment 14:**

Additionally, while the findings about Barishal district having **higher mitigation capacity are noted**, the factors contributing to this higher capacity (e.g., better infrastructure or preparedness) could be **explored more in-depth.**

**Response:**

We have expanded the results and discussion on Barishal district to provide a more in-depth analysis of the factors contributing to its higher mitigation capacity. Specifically, we now discuss the role of improved infrastructure, such as better road networks and communication systems, as well as enhanced preparedness measures, including early warning systems and community-based disaster response initiatives. These factors collectively contribute to Barishal's relatively higher capacity to mitigate risks compared to other regions. The updated analysis can be found in the revised manuscript on pages [15-19].

**Comment 15:**

The percentages and area calculations (e.g., "30.95% very high vulnerability", "26.42% moderate vulnerability") are useful, but they need clearer contextual explanation. A brief mention of how these areas were classified based on the vulnerability factors would add value. Were the classifications based on specific thresholds or ranges?

**Response:**

Thank you for your valuable feedback. We have added a clearer explanation of how the areas were classified based on vulnerability factors. Specifically, we have included details on the thresholds or ranges used to categorize vulnerability levels, such as the specific criteria or cut-off values for classifying areas as "very high," "moderate," and other levels of vulnerability. This additional context will help readers better understand the basis for the percentages and area calculations. The revised explanation can be found in the updated manuscript on pages [14-19].

**Comment 16:**

For **mitigation capacity**, the percentages are helpful, but more detailed descriptions of what "very low," "moderate," or "high" mitigation capacity entails in practice would provide better insights into the actual conditions on the ground.

**Response:**

Thank you for your insightful suggestion. We have expanded the description of "very low," "moderate," and "high" mitigation capacity in the manuscript to provide more concrete examples and context for each category. Specifically, we have clarified what each level of mitigation capacity entails in practice, including factors such as available resources, institutional support, and community preparedness. These additions aim to provide readers with a clearer understanding of the conditions on the ground. The updated descriptions can be found in the revised manuscript on pages 15-16 [lines 567-589].

**Comment 17:**

High resolution figures should be given.

**Response:**

High resolution figures have been uploaded to the Preflight Analysis and Conversion Engine (PACE) digital diagnostic tool.

We hope our responses and the revisions made to the manuscript address the reviewers’ concerns. Please do not hesitate to let us know if further clarification or modifications are required. Thank you for considering our revised manuscript.

Regards,

**Md Abdullah Salman**
