# Supplementary material for: Cyclone vulnerability assessment of the central coast of Bangladesh: A comprehensive study utilizing FAHP and geospatial techniques
Source: PLoS One. 2025 Jul 9;20(7):e0326965. doi: 10.1371/journal.pone.0326965 (PMC12240350; doi:10.1371/journal.pone.0326965)
Supplement: Attachment — Submitted filename: Response to Reviewers_(2nd Round).docx [file pone.0326965.s003.docx]

**Manuscript Title:** Cyclone vulnerability assessment of the central coast of Bangladesh: a comprehensive study utilizing FAHP and geospatial techniques

**Authors:** Md. Abdullah Salman^1*^, Mahmudul Hasan Rakib^1^, Shacin Chandra Saha^1^, Md Emdadul Haque^1^, Md Sabbir Hossen^1^

Journal Name: PLOS ONE

Manuscript ID: **PONE-D-24-47688**

**Dear Editor & [Reviewer],**

Thank you for your thorough review and constructive feedback on our manuscript. We appreciate the time and effort you dedicated to improving the quality of our work.

We have carefully considered all comments and suggestions. Below, we provide a point-by-point response to each comment. Revisions made to the manuscript are highlighted in yellow for clarity.

**Reviewer 01:**

**Comment 01:**

Limit the use of first-person pronouns (we used, we utilized, our study…etc.) across the whole manuscript.

**Response:**

Thank you for bringing this to our attention. We have replaced all the first-person pronouns and followed the entire manuscripts. You can find out on line 196 and 296.

**Comment 02:**

Line 123-125: You stated that “According to the literature, the Analytical Hierarchy Process (AHP) is the most prevalent and well-suited approach for spatial multi criteria evaluation [36].” Add that AHP is widly recognized in risk assessment associated with cyclones. Add a relevant reference such as “Mohseni, U., Jat, P. K. and Siriteja, V. (2025). Multi-criteria analysis-based mapping of the cyclone-induced pluvial flooding in coastal areas of India. DYSONA - Applied Science, 6(2), 309-321. <https://doi.org/10.30493/das.2025.490282>”

**Response:**

Thank you for highlighting the important reference. We have already added in the manuscript showing in line 132-134.

**Comment 03:**

Line 155-157: You stated that “Super Cyclones Sidr (2007) and Aila (2009) have severely damaged the coast, killing 3,500 people, injuring 191, and causing significant environmental and property damage [30].” Add that many still perceive them as a major cause of socioeconomic vulnerability and water scarcity in Southwestern Bangladesh. Add a relevant reference such as “Bhowmik, D., Kader, Z., Hosen, B. and Hossain, S. (2025). Exploring socioeconomic vulnerability and natural disasters impacts on water access in the south coastal region of Bangladesh. DYSONA - Applied Science, 6(1), 186-199. <https://doi.org/10.30493/das.2024.483646>”

**Response:**

Again, thank you for highlighting the important reference. We have already added in the manuscript highlighting in line 173-176.

**Comment 04:**

Fig. 2 is old fashion and somewhat mundane. Try making it more compact and modern adding some light coloring to it

**Response:**

Thank you for your valuable feedback regarding Figure 2. We appreciate your suggestion and have revised the figure accordingly. The updated version now features a more compact and modern layout with the addition of light, visually appealing colors to improve clarity and aesthetic quality. We believe this updated design enhances the overall readability and presentation of the information. Please see the revised Figure 2 in the updated manuscript.

**Comment 05:**

The results are well-presented and discussed. You also included a limitation section. However, you should discuss your view on how to overcome these limitation, and set few guidelines for future studies in light of your debates.

**Response:**

Thank you for acknowledging the presentation and discussion of the results, as well as the inclusion of the limitations section. In response to your insightful suggestion, we have expanded the discussion to address potential strategies for overcoming the identified limitations. Additionally, we have included a set of guidelines and recommendations for future studies, based on our findings and the challenges encountered in this research. These additions aim to provide a clearer path for advancing cyclone vulnerability assessments and improving methodological robustness in future work.

Please see the revised section in the updated manuscript (lines 709-772).

**Comment 06:**

Enhance the captions of figures and tables. A good caption should include all the data needed for a reader to comprehend the general purpose of figures and tables without returning to the text.

**Response:**

Thank you for your thoughtful suggestion regarding the figure and table captions. In response, we have revised and enhanced all captions to ensure they provide sufficient context and detail. The updated captions now clearly describe the content, purpose, and relevance of each figure and table, allowing readers to understand the key messages without referring back to the main text. These improvements aim to enhance clarity and improve the overall readability of the manuscript.

**Reviewer 02:**

**Comment 01:**

Justification of research: numerous studies have been conducted on cyclone vulnerability assessment in the region. It is better to state what is the new contribution from this study at the outset of this study.

**Response:**

Thank you for your valuable suggestion. In response, we have revised the introduction to clearly state the unique contributions of this study at the outset (lines 139-143). Specifically, this research focuses on the middle coastal zone of Bangladesh, a region that has received comparatively less attention in previous cyclone vulnerability studies. Additionally, the study integrates both physical and social vulnerability factors along with mitigation capacity using the Fuzzy Analytical Hierarchy Process (FAHP), which offers a more nuanced and accurate decision-making framework under uncertainty compared to traditional methods. These aspects provide a novel contribution to the existing body of literature on cyclone vulnerability assessment. The revisions have been incorporated to strengthen the justification of the study’s relevance and significance.

**Comment 02:**

Literature review and results: The study consults limited international literature of the discipline. Relevant examples can be drawn from the following recent sources in disaster prone settings:
• Change in cyclone disaster vulnerability and response in coastal Bangladesh
• Factors of cyclone disaster deaths in coastal Bangladesh,
• Reasons for non-evacuation and shelter-seeking behaviour of local population following cyclone warnings along the Bangladesh coast

**Response:**

Thank you for your insightful comment. In response, we have expanded the literature review to incorporate more relevant international literature, particularly the suggested recent studies that provide critical insights into human behavior and social dimensions of cyclone vulnerability (lines 101-108). Specifically, we have included the work of Paul (2020) on the changing patterns of cyclone disaster vulnerability and response in coastal Bangladesh, Paul and Dutt (2010) on factors influencing cyclone disaster deaths, and Alam and Collins (2010) on shelter-seeking and non-evacuation behavior. These studies have been integrated to strengthen the contextual foundation of our research and emphasize the importance of incorporating behavioral and socio-cultural factors into spatial vulnerability assessments. This inclusion not only enhances the comprehensiveness of the literature review but also aligns with the study’s aim of addressing both physical and social vulnerability in cyclone-prone regions.

**Comment 03:**

Since the results and discussion are presented together without a clear distinction, the interpretation of the data and the broader implications of the study remain ambiguous. This structure makes it difficult for readers to differentiate between the empirical findings and the authors' analytical insights or contextual explanations. As a result, the contribution of the study to existing knowledge, as well as its practical significance for cyclone vulnerability assessment and mitigation planning, is not effectively communicated. To enhance clarity and impact, the authors should consider separating the results from the discussion or, at a minimum, ensuring that each key finding is followed by a focused interpretation and explanation of its relevance within the broader research context. This would allow readers to better understand how the data supports the study’s conclusions and recommendations.

**Response:**

Thank you for this constructive observation. In response, we have revised the structure of the manuscript (separate Results & Discussion section) to enhance clarity and strengthen the analytical flow. Specifically, we have now clearly distinguished the results from the discussion by introducing dedicated sub-sections. Each key empirical finding is now presented in the results section, followed by a corresponding interpretation and contextual discussion in the discussion section. This structure not only allows for a clearer differentiation between data and analysis but also enhances the understanding of how the findings contribute to existing knowledge and inform cyclone vulnerability assessment and mitigation planning. We believe this adjustment significantly improves the readability, academic rigor, and practical relevance of the study.

**Comment 04:**

It is also less clear how the study will align with national and international development agendas.

**Response:**

Thank you for pointing out this important aspect. In response, the revised manuscript now includes a clarification on how the study aligns with both national and international development agendas (lines 800-806). Specifically, the study supports the goals of Bangladesh’s National Plan for Disaster Management (NPDM) by providing spatially detailed data for vulnerability assessment and risk-informed planning. At the international level, the research aligns with the Sendai Framework for Disaster Risk Reduction (2015–2030), particularly in its emphasis on understanding disaster risk, strengthening disaster risk governance, and enhancing disaster preparedness for effective response. Additionally, the study contributes to Sustainable Development Goals (SDGs), especially Goal 11 (Sustainable Cities and Communities) and Goal 13 (Climate Action), by offering insights that can help build resilience among vulnerable coastal populations. This connection is now clearly articulated in the revised discussion and conclusion sections.

**Comment 05:**

Conclusions seem to be just repeating some findings without linking the research findings with relevant disaster management policy and strategies in the region.

**Response:**

Thank you for your valuable observation. In response, the conclusion section has been revised to go beyond summarizing the findings by explicitly linking the research outcomes to relevant disaster management policies and strategies in the region (lines 776-811). Specifically, the revised conclusion highlights how the spatial vulnerability assessments can support targeted intervention planning, inform the prioritization of resource allocation, and guide the integration of both physical and social vulnerability considerations into national and local disaster preparedness frameworks. These policy-oriented insights aim to enhance the practical applicability of the study and contribute meaningfully to ongoing disaster risk reduction efforts in Bangladesh’s coastal regions.

We hope our responses and the revisions made to the manuscript address the reviewers’ concerns. Please do not hesitate to let us know if further clarification or modifications are required. Thank you for considering our revised manuscript.

Regards,

**Md Abdullah Salman**
